# Reduced blood flow and oxygenation in SA-1 tumours after electrochemotherapy with cisplatin

**DOI:** 10.1038/sj.bjc.6600606

**Published:** 2002-10-21

**Authors:** G Sersa, M Krzic, M Sentjurc, T Ivanusa, K Beravs, V Kotnik, A Coer, H M Swartz, M Cemazar

**Affiliations:** Laboratory of Radiation Biology, Institute of Oncology Ljubljana, Zaloska 2, SI-1000 Ljubljana, Slovenia; Jozef Stefan Institute, Jamova 39, SI-1001 Ljubljana, Slovenia; Veterinary Faculty, University of Ljubljana, Cesta v mestni log 47, SI-1000 Ljubljana, Slovenia; Institute of Microbiology and Immunology, Medical Faculty, University of Ljubljana, Zaloska 4, SI-1000 Ljubljana, Slovenia; Institute of Histology and Embryology, Medical Faculty, University of Ljubljana, Korytkova 2, SI-1000 Ljubljana, Slovenia; Department of Radiology, Dartmouth Medical School, Electron Paramagnetic Resonance Center for the Study of Viable Systems, Hanover, New Hampshire, NH 03755, USA

**Keywords:** fibrosarcoma, electroporation, cisplatin, electrochemotherapy, oxygen pressure, blood flow

## Abstract

Electrochemotherapy is an antitumour treatment that utilises locally delivered electric pulses to increase cytotoxicity of chemotherapeutic drugs. Besides increased drug delivery, application of electric pulses affects tumour blood flow. The aim of this study was to determine tumour blood flow modifying effects of electrochemotherapy with cisplatin, its effects on tumour oxygenation and to determine their relation to antitumour effectiveness. Electrochemotherapy of SA-1 subcutaneous tumours was performed by application of electric pulses to the tumours, following administration of cisplatin. Tumour blood flow modifying effects of electrochemotherapy were determined by measurement of tumour perfusion using the Patent blue staining technique, determination of tumour blood volume, and microvascular permeability using contrast enhanced magnetic resonance imaging, and tumour oxygenation using electron paramagnetic resonance oximetry. Antitumour effectiveness was determined by tumour growth delay and the extent of tumour necrosis and apoptosis. Tumour treatment by electrochemotherapy induced 9.4 days tumour growth delay. Tumour blood flow was reduced instantaneously and persisted for several days. This reduction in tumour blood flow was reflected in reduced tumour oxygenation. The maximal reduction in partial oxygen pressure (pO_2_) levels was observed at 2 h after the treatment, with steady recovery to the pretreatment level within 48 h. The reduced tumour blood flow and oxygenation correlated well with the extent of tumour necrosis and tumour cells apoptosis induced by electrochemotherapy with cisplatin. Therefore, the data indicate that antitumour effectiveness of electrochemotherapy is not only due to increased cytotoxicity of cisplatin due to electroporation of tumour cells, but also due to anti-vascular effect of electrochemotherapy, which resulted in reduced tumour blood flow and oxygenation.

*British Journal of Cancer* (2002) **87**, 1047–1054. doi:10.1038/sj.bjc.6600606 www.bjcancer.com

© 2002 Cancer Research UK

## 

Electroporation, i.e. application of electric pulses to the cells or tissues, induces under suitable conditions reversible permeabilization of cell membrane, thus facilitating entry into the cells of nonpermeant or poorly permeant molecules (Mir and Orlowski, 1999). Electroporation is currently used to facilitate cellular uptake of drugs, dyes, antibodies, and DNA (Mir, 2000). Some chemotherapeutic drugs are also poorly permeant for the cell membrane, but cytotoxic once reaching their intracellular targets. Cytotoxicity of these drugs can be increased by electroporation (Mir and Orlowski, 1999; Rols *et al*, 2000; Sersa, 2000a).

Electrochemotherapy consists of application of electric pulses to the tumours after drug administration. Increased cytotoxicity of bleomycin and cisplatin by electroporation was demonstrated *in vitro* on several rodent and human cell lines (Mir and Orlowski, 1999; Rols *et al*, 2000). In addition, good antitumour effectiveness of electrochemotherapy was demonstrated *in vivo* on different tumour models in animals (Sersa, 2000a). Electrochemotherapy with either bleomycin or cisplatin is effective at very low drug concentrations that without application of electric pulses, have minimal or no antitumour effect. Since the drug concentration used in electrochemotherapy is low, drug related side effects were not recorded so far. Furthermore, electrochemotherapy was effective in clinical settings. Several clinical trials on electrochemotherapy with either bleomycin or cisplatin have demonstrated its effectiveness in treatment of cutaneous and subcutaneous tumour nodules of head and neck tumours, basal cell carcinoma of the skin, malignant melanoma, and adenocarcinoma (Mir *et al*, 1998; Heller *et al*, 2000; Sersa *et al*, 2000b). From 70 to 100% objective responses were obtained 4 weeks after the treatment, with a high percentage of local tumour control.

Electroporation of the cells in tissue is apparently the basic principle of the antitumour effectiveness of electrochemotherapy. This was demonstrated by data on increased drug concentration in the tumours and increased binding of cisplatin to DNA after application of electric pulses (Belehradek *et al*, 1994; Cemazar *et al*, 1999). Furthermore, it was found that application of electric pulses to the tumours and normal tissues induces profound and reversible reduction in blood flow (Ramirez *et al*, 1998; Sersa *et al*, 1999a; Gehl and Geertsen, 2000; Gehl *et al*, 2002). Previous study has shown that tumour blood flow is further reduced in electrochemotherapy with bleomycin (Sersa *et al*, 1999b). In that study it was speculated that bleomycin after electroporation of tumours severely damages vasculature of the tumours and consequently induces a cascade of tumour cell death by ceasing oxygen supply to the cells. This presumption was supported by *in vitro* study, demonstrating that electroporation highly increases sensitivity of endothelial cells to bleomycin, whereas to cisplatin in lesser extent (Cemazar *et al*, 2001).

The aim of this study was to determine tumour blood flow modifying effects of electrochemotherapy with cisplatin, its effects on tumour oxygenation and to determine their relation to antitumour effectiveness. For this purpose contrast enhanced magnetic resonance imaging (MRI) and tumour staining with Patent blue were used, as well as electron paramagnetic resonance (EPR) oximetry.

## MATERIALS AND METHODS

### Drugs

cis-Diamminedichloroplatinum (II) (cisplatin) was obtained from Bristol Myers Squibb (Vienna, Austria) as a crystalline powder and dissolved in sterile H_2_O at a concentration of 1 mg ml^−1^. Final concentration was prepared in Eagle's minimal essential medium (EMEM, Sigma Chemical Co., St. Louis, MO, USA) for experiments *in vitro* or in 0.9% NaCl solution for experiments *in vivo*. For each experiment, fresh solution of cisplatin was prepared.

### Cells, tumours and animals

Murine fibrosarcoma SA-1 (The Jackson Laboratory, Bar Harbour, ME, USA) cells were used for the experiments. For the *in vitro* studies, they were grown in EMEM supplemented with 10% foetal calf serum (FCS; Sigma Chemicals St. Louis, MO, USA) in a humidified atmosphere at 37°C containing 5% CO_2_. The cells were routinely subcultured twice a week.

A/J mice, of both sexes, purchased from the Institute Rudjer Boskovic (Zagreb, Croatia) were used for this study. They were kept at constant room temperature (22°C) with a natural day/night light cycle in a conventional animal colony with food and water *ad libitum*. Before the experiments, the mice were subjected to an adaptation period of at least 10 days. The mice were 10–14 weeks old at the beginning of the experiments, weighing 20–25 g.

Tumour cells were obtained from the ascitic form of the tumours in mice, serially transplanted every 7 days. Solid subcutaneous tumours, located dorsolaterally or ventrally in mice, were initiated by an injection of 5×10^5^ SA-1 cells in 0.1 ml 0.9% NaCl solution. Six to 8 days after implantation, when tumours reached approximately 40 mm^3^ in volume mice were randomly divided into experimental groups. Treatment protocols were approved by the Department of Agriculture of the Republic of Slovenia No. 323-02-183/99. The ethical guidelines that were followed meet the standards required by the UKCCCR guidelines (Workman *et al*, 1998). For MRI and EPR oximetry, animals were anaesthetised with a mixture of Domitor (1.0 mg kg^−1^ body weight; Pfizer GmbH, Karlsruhe, Germany) and 10% ketamine (75.0 mg kg^−1^ body weight; Veyx-Pharma GmbH, Schwartzenborn, Germany) administered i.p. During anaesthesia, body temperature was kept at physiological values by a regulated heating pad on which the mice were placed (Guymar T/pump, Linton Instruments, Norfolk, UK).

### *In vitro* electrochemotherapy protocol

To determine the survival of SA-1 cells to combined treatment with cisplatin and electric pulses, 90 μl of a cell suspension (2.2×10^7^ cells ml^−1^) was mixed with 10 μl of different stock concentrations of cisplatin, as described previously (Sersa *et al*, 1995). One half of the mixture was exposed to eight electric pulses (1000 V cm^−1^, pulse duration 100 μs, frequency 1 Hz) and the other half served as a control for cisplatin treatment alone. The final cisplatin concentrations ranged from 0.5 to 400 μg ml^−1^. The cells were incubated for 5 min after electroporation, and thereafter clonogenic assay was performed (Sersa *et al*, 1995). The survival of cells treated with combination of electric pulses and cisplatin was normalized to electric pulse treatment alone. The surviving fraction of cells treated with electric pulses alone was 0.85±0.1 (data not shown). The experiments were performed twice, experimental groups in triplicates.

### *In vivo* electrochemotherapy protocol

Cisplatin at a dose of 4 mg kg^−1^ was injected intravenously. Injection volume was 7.5 ml kg^−1^ body weight. Eight square-wave electric pulses, delivered in two sets of four pulses in perpendicular directions, of 1040 V amplitude (1300 V cm^−1^), with pulse width of 100 μs and repetition frequency 1 Hz were delivered by two flat, parallel stainless-steel electrodes 8 mm apart (two stainless-steel strips: length 15 mm, width 7 mm with rounded corners), which were placed percutaneously at the opposite margins of the tumour. Good contact between the electrodes and the skin was assured by means of conductive gel. Electric pulses were generated by an electroporator Jouan GHT 1287 (Saint Herblaine, France). In the electrochemotherapy protocol, mice were treated with electric pulses 3 min after cisplatin injection. All treatments were performed without anaesthesia and were well tolerated by the animals (Sersa *et al*, 1995).

Tumour growth was followed by measuring three mutually orthogonal tumour diameters (e_1_, e_2_ and e_3_) with a vernier caliper three times per week. Tumour volumes were calculated by the formula *V*=π×e_1_×e_2_×e_3_ /6. The arithmetic mean of tumour volumes and standard error of the mean (s.e.) were calculated for each experimental group. Tumour doubling time was determined for each individual tumour from the growth curves. Tumour growth delay was calculated for each individual tumour by subtracting the doubling time of each tumour from the mean doubling time of the control group and then averaged for each experimental group.

### Assessment of tumour perfusion by Patent blue staining

Patent blue (Byk Gulden, Konstanz, Germany) was used to estimate tumour perfusion. Patent blue (1.25 %) was injected (0.2 ml) at different time points after treatment into the tail vein of animals with tumours from control, electric pulses, cisplatin and electrochemotherapy groups. After the dye was distributed evenly through the tissue (1 min), animals were killed by cervical dislocation which results in instantaneous death of the animal and tumours excised, carefully removed from the skin, cut in half along their largest diameter, and immediately evaluated. The percentage of stained area for each of the faces of the two halves of tumour cross-section (perfused), as opposed to nonstained area (nonperfused) was visually estimated by three individuals in blind fashion. The mean of both estimations was used as an indicator of tumour perfusion. Because of marked differences in tumour staining between well-perfused and poorly perfused tumours, visual estimation of the whole tumour cross-sections gives good estimation and reliable results. In the study comparing Patent blue staining and the pharmacological method (^86^RbCl extraction technique) measuring relative tumour blood flow, good correlation between both methods was found (*r*=0.962) (Sersa *et al*, 1999b). Briefly, ^86^RbCl extraction technique gives measure of tissue perfusion as a function of cardiac output and is expressed as percent injected activity per gram or relative uptake (per cent control) (Sapistein, 1958).

### Dynamic contrast-enhanced MRI

An intermediate molecular size macromolecular magnetic resonance contrast agent gadomer-17 (Shering AG, Berlin, Germany) was used to assess changes in fractional tumour blood volume (BV) and microvascular permeability surface area product (PS) in control, electric pulses, cisplatin and electrochemotherapy treated animals. When administered i.v., this agent does not diffuse through endothelium, unless there is a pathological change (Demsar *et al*, 1997; Beravs *et al*, 2000). The size of the agent is approximately 30 kDa, facilitating its complete renal elimination. The agent was administered in a dose of 0.025 mmol kg^−1^ in a bolus via 23-gauge intravenous cannula inserted into a tail vein of anaesthetised mice. No systemic toxicity was observed. Animals were placed in the 2.35 T Bruker Biospec system with a horizontal bore magnet.

A pre-contrast image (complete k-space data set) was acquired first, using a standard spin-echo technique. Imaging parameters were: repetition time, T_R_=600 ms; echo time T_E_=18 ms; matrix, 256×256; slice thickness, 2 mm; field of view, 7 cm; and acquisition time 5 min. Second, gadomer-17 was administered, and a central data subset of the k domain (in the phase-encoding direction) with dimensions 32×256 k space data points was acquired repetitively for 70 min (80–100 ‘key’ images). Each key image was acquired with 32 phase-encoding steps that took 38 s. Before the reconstruction, the subset was first completed in remaining k-space points (which were not included in temporal acquisition) with the data from the pre-contrast acquisition. Finally, images were reconstructed with two-dimensional inverse Fourier transformation.

To obtain a measure for altered blood flow (PS and BV), the magnetic resonance signal was measured within the tumour and vena cava in pre-contrast image and at least 80 post-contrast images that were acquired every 38 s. The signal was corrected for signal variations against a water phantom. From the measured signal, the concentration *C_T_*, of the gadomer-17 in the tumour was calculated by subtraction of the pre-contrast signal from the post-contrast signals on a pixel-by-pixel basis. The T_1_ relaxivity in water Gd^−1^ at 20 MHz and 39 °C is 14.4 mmols^−1^ (Demsar *et al*, 1997). The concentration of gadomer-17 in a slow-flowing vessel, such as the inferior vena cava, *C_B_*, was obtained in a similar way. The linearity of the *C_T_/C_B_* fit was checked for the first 30–50 points. *PS* and *BV* were calculated using an established method (Demsar *et al*, 1997). *PS,* which represents fraction of contrast agent leaking out of the blood in time [% h^−1^] was calculated as:


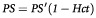


where *Hct* is the measured haematocrit of the blood (47% for tumours in animals) and *PS′* is linear fit coefficient of *C_T_/C_B_* at different time points. *BV*, which represents fraction of contrast agent leaking through tumour endothelium into interstitial space in time was calculated as:


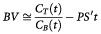


where *C_T_* is concentration of contrast agent in tumour and *C_B_* concentration of contrast agent in blood.

### EPR oximetry

EPR oximetry was used to evaluate changes in partial oxygen pressure (pO_2_) in tumours and subcutis on the contralateral side before and after treatment of tumours with electric pulses, cisplatin and electrochemotherapy. EPR oximetry is a non-invasive method, (after insertion of the paramagnetic probe) which allows monitoring of pO_2_ repeatedly at the same point in the tissue over long periods of time (O'Hara *et al*, 2001; Sersa *et al*, 2001). For this purpose a paramagnetic probe, a char (0.5 mm^3^, with particle size 10 μm) made from Bubinga tree (a kind gift of EPR centre for viable system, Dartmouth College of Medicine, Hanover, NH, USA), which is sensitive to oxygen was implanted into tumour center and periphery (tumour volume approximately 40 mm^3^) and subcutis one day before the treatments and its EPR spectral line-width measured at different time points up to two days after treatment (Krzic *et al*, 2001). The char implantation with a sterile needle did not provoke bleeding. In the presence of oxygen, the line-width of the EPR lines is broadened; the extent of broadening depends on pO_2_ (Swartz *et al*, 1993; Swartz and Clarkson, 1998). The pO_2_ in the region in contact with the probe was measured.

The measurements were performed on a Varian E-9 EPR spectrometer, with a custom-made low-frequency microwave bridge operating at 1.1 GHz and an extended loop resonator (11 mm in diameter), both designed by Professor T Walczak (Dartmouth Medical School, Hanover, NH). Typical spectrometer settings were: modulation frequency, 100 KHz; modulation amplitude not more than one-third of the peak-to-peak line-width, and scan range, 2 mT. The line width of the EPR spectra reflects the pO_2_, which was determined from an existing calibration curve (Krzic *et al*, 2001).

### Histology of tumours

The same tumours that were used for Patent blue staining were fixed in 10% buffered neutral formalin. Sections through the largest diameter of the tumours were embedded in paraffin, cut and stained with haematoxylin and eosin by standard method. Necrosis in slides of 3–9 tumours per group were determined in blind fashion by three experts. The percentage of necrotic area was determined in whole mount tumour sections.

The apoptotic rate was determined using Cell Death Detection ELISA (Boehringer Mannheim, Germany). The assay is based on the quantitative sandwich-enzyme-immunoassay principle and detects apoptotic cells relatively selectively even in the presence of an excess of necrotic cells (Leist *et al*, 1995). This procedure permits a specific determination of mono and oligonucleosomes in the cytoplasmic fraction of cell lysates characteristic for apoptosis. Tumours used for determination of apoptosis were first squeezed between two sintered microscope slides. Then the suspension was washed and cells exposed to the lysing buffer. After lysis, supernatant containing apoptotic bodies was prepared by centrifuging the sample at 15 000 r.p.m. In the first step of ELISA procedure, monoclonal anti-histone antibodies directed against H2A, H2B, H3 and H4 histone antigens were fixed to the wall and the bottom of the microtiter plate module. During the second step, the nucleosomes contained in the sample bind via their histone components to the immobilised anti-histone antibodies. In the third step, anti-DNA-POD antibodies reacted with the DNA part of the nucleosome. The amount of peroxidase retained in the immunocomplex was determined spectrophotometrically with ABTS (2,2′-azino-di-(3-ethylbenzthiazoline sulphonate)), as substrate. All the measurements were done in triplicates on six mice per group.

### Statistical analysis

All data were tested for normality of distribution using the Kolmogrov–Smirnov test. The differences between the mean values of groups were tested for significance by a *t*-test after one-way ANOVA was performed and fulfilled. SigmaStat statistical software (SPSS inc.) was used for statistical analysis. *P* levels of less than 0.05 were taken as significant.

## RESULTS

### Antitumour effectiveness

**Figure 1:**
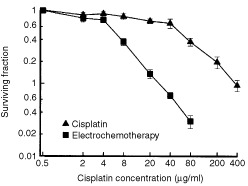
Potentiation of cisplatin cytotoxicity *in vitro* by electroporation of SA-1 tumour cells. The clonogenicity of cells was determined after cisplatin treatment for 5 min, exposure to electric pulses, or combination of both (electrochemotherapy). The survival curve for the electrochemotherapy treated cells was normalised for the cytotoxicity of electric pulses treatment alone. Surviving fraction of cells treated with electric pulses alone was 0.85±0.1. Symbols represent mean values±s.e. of pooled data from two experiments done in triplicates.

The cytotoxicity of cisplatin after electroporation of fibrosarcoma SA-1 cells was determined *in vitro* by a colony forming assay. Exposure of cells to electric pulses resulted in a marked increase in cisplatin cytotoxicity. Throughout the range of cisplatin concentrations investigated, cells exposed to electric pulses were more sensitive to cisplatin than those that were not (Figure 1

). The cells exposed to electric pulses were 10-fold more sensitive to cisplatin as determined at the concentration causing 50% inhibition of colony formation.

Treatment of subcutaneous SA-1 tumours by electrochemotherapy with cisplatin was very effective, resulting in substantial tumour growth delay, compared to untreated tumours and tumours treated with cisplatin or application of electric pulses only (Table 1

**Table 1:**
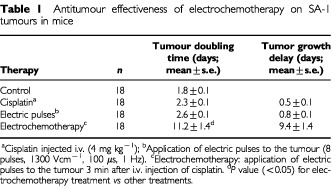
Antitumour effectiveness of electrochemotherapy on SA-1 tumours in mice

). Electrochemotherapy treatment induced a reduction of tumour volume that was observable 3 days after the treatment. Thereafter the tumours gradually reduced in size and by the 6th day they had decreased to pretreatment values. Tumours were neither ulcerated nor had a superficial scab. Regrowth of tumours started at the 7th day after the treatment. Their growth rate then was approximately the same as in the control groups. The treatment of animals with cisplatin or application of electric pulses to the tumours as single treatment did not result in a substantial antitumour effect.

### Tumour blood flow

**Figure 2:**
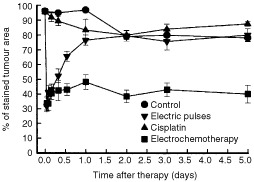
Time course of changes in tumour perfusion measured by Patent blue staining in untreated control tumours, after treatment of tumours by application of electric pulses, cisplatin, or electrochemotherapy. Symbols indicate mean values±s.e. of at least six mice per point.

The time course of tumour perfusion changes was evaluated in untreated, tumours treated by cisplatin or application of electric pulses only, and after electrochemotherapy. Application of electric pulses to the tumours induced a significant and rapid reduction in tumour perfusion as measured by Patent blue staining (Figure 2

). Within 2 h tumour perfusion was reduced to 30%, but returned to near the pretreatment value within 24 h. Thereafter the perfusion was similar to the perfusion in untreated or cisplatin treated tumours. Electrochemotherapy induced the same onset of tumour perfusion changes as the application of electric pulses. Thereafter, within 8 h tumour perfusion was restored to approximately 40% of the pretreatment value where it remained up to the 5th day. From the 8 h to the 5th day after the treatment with electrochemotherapy, tumours were significantly less perfused than tumours treated with cisplatin or electric pulses only (Figure 2).

**Figure 3:**
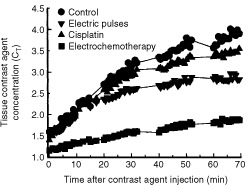
Time course of gadomer-17 accumulation in untreated control tumours and tumours 24 h after treatment with cisplatin, application of electric pulses, and electrochemotherapy. Two tumours were implanted ventrally in mice. In one set of experiments one tumour served as control, to the other electric pulses were applied. In the other set of experiments mice were injected with cisplatin and one tumour also received electric pulses (electrochemotherapy). Contrast agent was injected at time zero, each group consisted of three mice; for simplicity error bars were omitted.

Dynamic contrast-enhanced MRI was used to evaluate changes in tumour BV and PS 24 h after treatment with cisplatin, application of electric pulses and after electrochemotherapy. The tumour periphery was enhanced more than the centre of the tumour in the first minute after the injection of the contrast agent, indicating that the periphery was better vascularised than the centre (data not shown). The tumour enhancement increased gradually, indicating diffusion of the contrast agent from the blood into the interstitial space. Tumours treated with electrochemotherapy showed delayed enhancement with gadomer-17, indicating a change in blood flow or decreased permeability. The concentration of contrast agent in the tumour (*C_T_*) gradually increased over 70 min observation time (Figure 3

). The smallest reduction in contrast agent accumulation was observed after treatment with cisplatin. Application of electric pulses caused a moderate reduction in contrast agent accumulation, which correlated with the observations of tumour perfusion after Patent blue staining. Electrochemotherapy resulted in a larger (2.5-fold) reduction in contrast agent accumulation. These observations were supported by calculated tumour BV and PS (Table 2

**Table 2:**
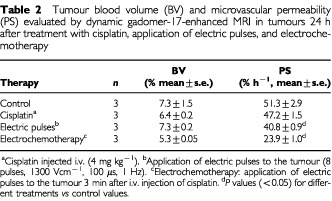
Tumour blood volume (BV) and microvascular permeability (PS) evaluated by dynamic gadomer-17-enhanced MRI in tumours 24 h after treatment with cisplatin, application of electric pulses, and electrochemotherapy

).

### Tissue oxygenation

EPR oximetry was used to measure pO_2_ in the tumours as well as in subcutis. The pO_2_ values in normal subcutis (8.37±0.05 mmHg) were higher than in the untreated tumour tissue (Table 3

**Table 3:**
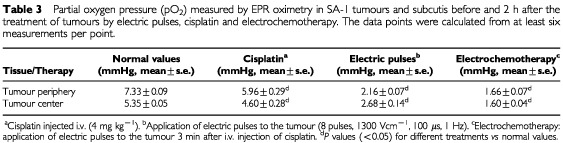
Partial oxygen pressure (pO_2_) measured by EPR oximetry in SA-1 tumours and subcutis before and 2 h after the treatment of tumours by electric pulses, cisplatin and electrochemotherapy. The data points were calculated from at least six measurements per point

). Within the untreated tumours, higher pO_2_ values were measured in the tumour periphery (7.3 mmHg) than in the tumour centre (5.3 mmHg).

**Figure 4:**
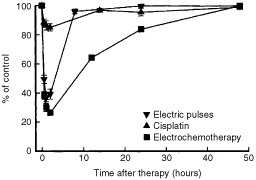
Time course of changes in partial oxygen pressure (pO_2_) measured by EPR oximetry in tumours (combined data for tumour centre and tumour periphery) after the treatment of tumours by application of electric pulses, cisplatin, or electrochemotherapy. Symbols indicate mean values±s.e. of six mice per point.

The time course of pO_2_ changes in tumours was similar to the changes in tumour perfusion, as measured by Patent blue staining. A very fast and profound reduction of pO_2_ was observed in the tumours after application of electric pulses to the tumours and after electrochemotherapy. The maximal reduction was observed within 2 h after the treatment, with steady recovery thereafter. The recovery to pretreatment levels was slower after electrochemotherapy (48 h) than after application of electric pulses (8 h). Treatment with cisplatin only and effects on subcutis, contralateral to the application of electric pulses or electrochemotherapy had only short lasting effects on pO_2_ (Table 3; Figure 4

). Effects of different treatments; cisplatin, electric pulses and electrochemotherapy had only short lasting effects on pO_2_ in the contralateral subcutis, with maximal reduction at 2h (7.35±0.13; 7.66±0.08; 6.80±0.1 mmHg, respectively).

### Necrosis and apoptosis

**Figure 5:**
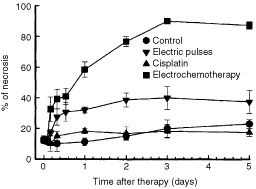
Time course of changes in tumour necrosis in untreated control tumours, and after treatment of tumours by application of electric pulses, cisplatin, or electrochemotherapy. Symbols indicate mean values±s.e. of at least three mice per point.

**Figure 6:**
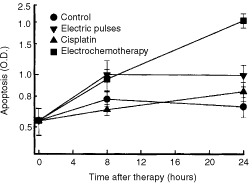
Time course of changes in apoptotis (O.D.) in the untreated control tumours, and tumours after treatment by application of electric pulses, cisplatin, or electrochemotherapy. Symbols indicate mean values±s.e. of six mice per point.

The time course of tumour necrosis was evaluated for 5 days after treatment. The extent of necrosis in the cisplatin treated tumours was similar to the untreated tumours. Application of electric pulses to the tumours induced a slow increase in tumour necrosis up to 8 h after treatment, and remained on this level up to the 5th day. In electrochemotherapy treated tumours the degree of tumour necrosis followed the onset of tumour necrosis in the group treated with electric pulses only, and continued to increase up to the 3rd day after the treatment, when it reached 90% and it remained at this level through the 5th day (Figure 5

). In the tumours treated with electrochemotherapy irreversible cell injury also was shown by confluent areas of cells with distinct changes. Tumour cells were swollen with picnotic nuclei. Perinuclear cytoplasm was condensed, eosinophilic, and filled with multiple blebs at the periphery of the cells. In electrochemotherapy treated tumours, in non-necrotic tumour areas distinct apoptotic cells and apoptotic bodies were noticed. Electrochemotherapy induced apoptosis in the tumours was confirmed by an ELISA assay. A statistically significant fourfold increase in apoptosis was observed 24 h after the treatment with electrochemotherapy compared to untreated controls (Figure 6

).

## DISCUSSION

This study shows that electrochemotherapy with cisplatin reduces tumour blood flow instantaneously and persists for several days. This reduction in tumour blood flow was reflected in reduced tumour oxygenation recovering to the pretreatment level within two days. The time course of reduced tumour blood flow and tumour oxygenation correlated with time course of antitumour effectiveness, tumour necrosis and apoptosis induced by electrochemotherapy with cisplatin. Therefore, the data indicate that antitumour effectiveness of electrochemotherapy is not only due to increased cytotoxicity of cisplatin due to electroporation of tumour cells, but also due to anti-vascular effect of electrochemotherapy, which results in reduced tumour blood flow and oxygenation.

Increased cytotoxicity of the drugs by electroporation is the predominant mechanism involved in antitumour effectiveness of electrochemotherapy. This notion is supported by the data *in vitro* demonstrating that exposure of tumour cells to electric pulses increases cytotoxicity of bleomycin several 100-fold, and as in this study, cisplatin cytotoxicity 10-fold (Jaroszeski *et al*, 2000). Among several other known anti-neoplastic drugs that were tested, these two drugs were shown to be the best candidates for electrochemotherapy, because they are hydrophilic and have good antitumour effectiveness once their access to cytosol is facilitated by electroporation (Sersa *et al*, 1995; Mir and Orlowski 1999). Presumption, that increased cytotoxicity of the drugs is the main mechanism involved in antitumour effectiveness of electrochemotherapy, is supported by the data demonstrating that electroporation causes increased bleomycin as well as cisplatin accumulation in the tumours (Belehradek *et al*, 1994; Cemazar *et al*, 1999). Specifically, in SA-1 tumours, as used in this study, the amount of platinum bound to DNA and platinum content in the tumours treated by electrochemotherapy was approximately two times higher compared to cisplatin treatment alone (Cemazar *et al*, 1999). All these data support the notion that electroporation either *in vitro* or *in vivo* is the predominant mechanism involved in antitumour effectiveness of electrochemotherapy.

In view of our previous study, demonstrating that electrochemotherapy with cisplatin induced more than a 20-fold increase in cell kill in SA-1 tumours, compared with cisplatin treatment alone, we proposed that, in addition to electroporation of tumour cells that induces approximately 10-fold increase in cytotoxicity in SA-1 cells *in vitro*, other mechanism may be involved in antitumour effectiveness of electrochemotherapy (Cemazar *et al*, 1999). Additional mechanisms involved in antitumour effectiveness of electrochemotherapy with cisplatin were proposed: immune response (Mir *et al*, 1992; Sersa *et al*, 1997), anti-vascular effect due to cytotoxic action on endothelial cells (Cemazar *et al*, 2001) and tumour blood modifying effect of electrochemotherapy (Sersa *et al*, 1999b).

Electroporation is a universal process, therefore application of electric pulses to solid tumours would be expected to increase permeability of cell membrane of all cells that are treated. Potential targets include endothelial cells lining the tumour vasculature. Damage to endothelial cells may lead to obstruction of blood flow and ischaemic death of tumour cells lining the obstructed blood vessel. This phenomenon, described as anti-vascular or vascular-targeted therapy, gained high interest and has been exploited in several studies (Chaplin *et al*, 1998; Denekamp *et al*, 1983). In the first step to confirm that electrochemotherapy has an anti-vascular effect, we determined in our previous study sensitivity of human microvascular endothelial cells (HMEC-1) to bleomycin and cisplatin, with and without *in vitro* electroporation of cells (Cemazar *et al*, 2001). HMEC-1 cells were moderately sensitive to continuous exposure to cisplatin, but showed greater sensitivity to bleomycin. However, electroporation of cells increased cytotoxicity by ∼10-fold for cisplatin and ∼5000-fold for bleomycin.

The above mentioned is supported by the observations that electrochemotherapy with bleomycin resulted in complete shut down of tumour blood flow observed by 12 h after the treatment, and correlated with antitumour effectiveness and the extent of tumour necrosis (Sersa *et al*, 1999b). In the present study, the antitumour effectiveness of electrochemotherapy with cisplatin could also in part be ascribed to its anti-vascular effect. Electrochemotherapy induced significant reduction in tumour perfusion, stabilizing at 40% pretreatment level up to the 5th day after treatment. The data on contrast enhanced MRI support these observations, 24 h after the electrochemotherapy tumours were significantly less enhanced than after the treatment with cisplatin or electric pulses.

Reduced tumour blood flow is expected to result in lower oxygen tension. By using EPR oximetry we were able to follow the time course of oxygen tension in the tumours. Electrochemotherapy induced profound and long lasting pO_2_ reduction, which is expected to cause some additional cell kill in the tumours due to the prolonged hypoxia. The onset and time course of tumour oxygenation changes correlated with tumour blood flow reduction, and resulted in gradual increase in extent of tumour necrosis, that reached approximately 90% of the tumour area, three days after treatment. That coincided with the time when the tumours started to reduce in the size and ceased to grow. The tumours started to regrow only 7 days after the treatment. The resulting antitumour effect was 9.4 days tumour growth delay, which is significantly better than the effect of single treatments, cisplatin (0.5 days tumour growth delay) and application of electric pulses (0.8 days tumour growth delay). The effect was less pronounced than in electrochemotherapy with bleomycin, which is consistent with *in vitro* data on endothelial cells sensitivity (Sersa *et al*, 1999b; Cemazar *et al*, 2001).

The proportion of apoptotic cells increased only after electrochemotherapy with cisplatin. Cisplatin is known to induce apoptotic cell death, therefore it can be speculated that electrochemotherapy, which increases cisplatin delivery into the cells, also stimulates apoptotic cell death (Meyn *et al*, 1995; Jordan and Carmo-Foncesca, 2000). In histological sections, apoptotic cells were observed in non-necrotic regions, however necrotic regions represented a major part in the sections of tumors treated with electrochemotherapy, demonstrating that necrosis is a dominant way of cell death induced by electrochemotherapy. In tumours treated with cisplatin only apoptosis was not observed in the histological sections and also not detected by use of ELISA test for determination of mono and oligonucleosomes in the cytoplasmic fraction of cell lysates. The cisplatin dose used in experiments was very low and is most probably the reason for the absence of apoptosis. However, further studies are needed to fully understand the mechanisms of cell death induced by electrochemotherapy with cisplatin. It could be that higher amounts of cisplatin in the cells combined with electroporation might more efficiently trigger signalling pathways that lead to cell death. When response of the tumours to electrochemotherapy using bleomycin or cisplatin is compared macroscopically, tumours after electrochemotherapy with cisplatin respond to the treatment much slower and usually there is no ulceration of the tumours.

In conclusion, this study provides data on physiological changes in the tumours, as a consequence of electrochemotherapy with cisplatin and correlates these effects with antitumour effectiveness and histological changes in the tumours. The results indicate that reduction in tumour blood flow and tumour oxygenation correlate with extensive tumour necrosis and induction of apoptosis of the tumours treated by electrochemotherapy with cisplatin. The data support the evidence that the antitumour effectiveness of electrochemotherapy is not only due to increased drug delivery to the cells, but also to anti-vascular effect of electrochemotherapy, which results in reduced tumour blood flow and oxygenation.
